# Medical and Philosophical Intelligence

**Published:** 1819-09

**Authors:** 


### Medical and Philosophical Intelligence.

### NOTICE OF SUCH OF THE TRANSACTIONS OF PHILOSOPHICAL SOCIETIES, AS RELATE TO MEDICINE AND ITS AUXILIARY SCIENCES.

*ROYAL SOCIETY of LONDON.* July 1st.—A PAPER was read by ARTHUR JACOB, M.D. demonstrator of anatomy at Trinity-college, Dublin, giving an account of his discovery of a new membrane in the eye, covering the external surface of the retina, and united to it by cellular substance. It may be displayed by cutting off the sclerotica and choroides, and looking on the retina in water, when it will be seen floating above the surface of the retina.

AT a sitting of the *Faculty of Paris* on the 6th of May, Professor DUPUYTREN mentioned having a patient under treatment, on whom he was employing compression for the cure of a wound of the femoral artery by a stroke from a knife. He mentioned several facts that have occurred to his observation, which lead him to conclude, contrary to the opinion generally adopted, that wounds of arteries may be cured without either the obliteration of their cavity, or the subsequent formation of aneurism. He has presented a patient with a rupture of the femoral artery as it crosses the third adductor muscle, whom he also intends to treat by his plan of compression.

M. Dupuytren, a short time since, tied the subclavian artery at the situation of its passage across the scalenus muscle, by making an incision in the anterior part of this muscle, for a false aneurism of the vessel. His motives for tying the artery in this situation rather than in its course between this muscle and the clavicle, were, that in the former point the vessel is completely separate from the vein and the nerves of the brachial plexus, whilst in the second it is confounded with those parts, and can only with difficulty be separated from them. This case terminated favourably.

M. Fol, a surgeon of Vandœuvres, near Geneva, has recently published* *a case of Tetanus*, that terminated favourably under the use of mercurial frictions, jalap, and narcotics exhibited internally and applied externally. The patient was a stout and healthy common labourer, 27 years of age: the disease originated from the nail of the right thumb being nearly torn off. In somewhat less than a month after the accident the symptoms of tetanus appeared. He had, Oct. 5th, frequent and severe rigors; great stiffness of all the voluntary muscles; those of his jaw were painfully contracted; and the abdomen was very tense. The pulse on the preceding evening was languid, and some apprehension was formed that the heart was somewhat affected. On this day, the 5th, the pulse was more developed, but still contracted. A dose, composed of three grains of calomel, fifteen grains of anodyne powder,† three grains of jalap, and twelve grains of salt of amber, was given every two hours. Frictions with mercurial ointment were made along the spine, the body generally rubbed with oil, and to the abdomen were applied warm fomentations. Glysters of assafœtida and watery solution of opium, were given every four hours. Fumigations of opium and salt of amber were also employed: this was effected by burning under the bed-clothing, on lighted wood in a proper machine, a powder composed of half a drachm of salt of amber and fifteen grains of opium. The disease was almost entirely relieved in five days, and finally totally disappeared.

Dr. Firth, of Hyde-park, South-Carolina, has cured a case of the same disease by the use of opium, cantharides, a decoction of *asarum canadense* as common drink, with the warm-bath every three hours.‡ The patient was a female negro: the disease seemed to originate from a wound by a nail in the right *malleolus externus*. The disease appeared on the day preceding that on which Dr. Firth first saw her. "Her jaws were now fixed; and every half-hour she had a violent attack of opisthotonos." He scarified the punctured ankle very deep, "so as to divide any of the nervous branches which might be injured; poured in spirit of turpentine, and applied a dossil of lint impregnated well therewith, over which was placed a poultice." A drachm of tincture of cantharides, and the same quantity of tincture of assafœtida, was given every three hours. The disease was somewhat alleviated. Perfect relaxation of the jaw took place whilst the patient was in the bath, but the contraction returned on leaving it. Blisters were then applied to the temporal muscles, nape of the neck, and the spine of the back; she was purged with calomel and gamboge, and had glysters given her. "I observed," says Dr. F. "that, whenever the blisters discharged freely and strangury existed, the disease was moderated, the spasms occurred but seldom, and the jaws were relaxed: when the strangury subsided, the disease increased in violence. The following was then ordered:

---

* In the *Journal Universel des Sciences Medicales*, July 1819.

† We regret that we are not able to give the exact composition of this; but it contains a considerable proportion of opium.—Edit.

‡ This case is related in the *New-York Medical Repository*, April 1819.

Take, tinct. cantharid. . . . ℥ iij ſs.

—— opii . . . . . ℥ iij ſs.

Sp. lavend. comp. . . ℥ ij M.

To take one table-spoonful five times in the twenty-four hours. The jaws were rubbed five times a-day with a lump of mercurial ointment, the size of a nutmeg.

She recovered in a few days, though a stiffness of the jaw remained for some time longer; but which disappeared on salivation taking place.

---

The attending physicians of the New-York Dispensary, Doctors Dyckmann, Cheesman, Cockroft, and Townsend, accompanied by Doctors Aydelott and Ducachet, had an opportunity, during the last month, of verifying the curious discovery, by Prof. Richerand, of the *transparency of the pericardium;* which they find to be true in the dead as well as in the living subject. The case occurred in the examination of a mulatto-child of two years of age, twelve hours after death. The child had died of the febrile remittent of infants: there were two or three ounces of colourless water in the pericardium, a considerable quantity in the brain, and the body was yet warm.

The transparency observed in the above subject has, no doubt, existed as long as the warmth of the body.—*New-York Medical Repository*, April 1819.

---

M. Bouillon-Lagrange has given, in the *Journal de Pharmacie*, June 1819, the *composition of a topical application for indurated tumors in the female breast*, which he has found remarkably efficacious. Several cases, threatening to appear in a cancerous character, have been much, or even totally, removed by it. It acts, he says, with a singular influence on the exhalents of the skin; from which a large quantity of reddish-coloured viscous secretion takes place, "soon producing the solution and disappearance of the most voluminous and hard tumefaction of the glands." It is as follows:

Take, simple plaster (litharge plaster) four ounces; melt it with a gentle heat in an earthen vessel, and add one ounce of yellow wax, half an ounce of white soap, and four drachms of turpentine: these are to be melted together, then removed from the fire, and two drachms of powdered cicuta, two drachms of powdered sulphuret of potash, and four drachms of powdered camphor, then added. To be well mingled, and made into rolls.

---

The use of *animal magnetism* has been prohibited in all the provinces of the Austrian empire: a public censure has been passed on those physicians who had encouraged the delusion of its application; and they are interdicted from again employing it, under the penalty of suspension of their functions. Such a measure had become highly necessary, for it seemed to be recently gaining ground to a serious extent. Many physicians of high character encourage it at the present time in Germany; and there is a periodical journal (*Archiv für den thiereschen Magnetismus*, published at Halle, by Drs. Escherrmayer, Kieler, and Nasse,) instituted for the purpose of recording their practice and opinions.

### REPORT OF DISEASES.

WE have to repeat, in the present Report, the remark made in the three or four preceding ones, that the present state of the inhabitants of the metropolis is particularly healthy. No affection is so prevalent as to bear at all the appellation of an epidemic, and there does not exist any general and severe form of disease. The present, and the two or three subsequent months, are indeed generally the most salubrious to the inhabitants, taking into the calculation of the number of patients, the comparative emptiness of the city, and the consideration that those who have now left it form a great proportion of the number of invalids, wherever they may be present.

The most common acute disease is diarrhœa, not often accompanied with much, or even any, fever. This has generally soon subsided, under a regimen of restriction from all solid and irritating food, with the use of rhubarb, ipecacuanha, and occasionally small doses of opium and the absorbent earths. Some cases have been most effectually relieved by saline purgatives; and others, by small doses of tartar emetic. The most severe cases have been those that have in the first instance, as is very common with the vulgar, been treated by cordials, or large doses of opiates and astringents. One instance of this kind, in a previously healthy middle-aged man, has been followed by remittent fever, occurring in paroxysms accompanied by pain in the intestines, followed by mucous stools.

Pneumonic inflammation is not an uncommon occurrence; sometimes without much fever, at others with affection of the brain and the usual symptoms of typhus. The latter has appeared chiefly in either the lower classes of people, those of intemperate habits, or when the disease has been mismanaged in its first stage. It is lamentable to witness the fear of blood-letting entertained by some practitioners, because what is commonly termed *typhoid* debility may be present, notwithstanding the practical evidence that has been accumulating during the last century, especially, to show its necessity; and the satisfactory doctrine that such debility is, when the patient has previously to the attack been in health, either partial from a concentration of vital action in some organ, or the necessary consequence of a compressed brain; as Dr. KINGLAKE has well explained in the present number of this Journal.

Cutaneous eruptions (chiefly *ecthyma* and *eczema*) are common in children, evidently forming one symptom of a train of morbid affections originating in gastric disorder, and commonly appearing suddenly, and suffering exacerbations and remissions, subsequently to internal disease, and attacks of febricula.

Peritoneal inflammation, which we mentioned to be rather prevalent, has suddenly disappeared from the list of maladies. It seems to be changed for affections of the mucous membranes.

Rheumatism is one of the most distressing of the chronic complaints, and the form of it that occurs especially at this season is that which most obstinately resists medical treatment. The patients of it very frequently have been victims to various other disorders, sometimes of an anomalous character, and are commonly in a debilitated state. Perhaps the rheumatic affection should not be much regarded in the treatment.

### METEOROLOGICAL JOURNAL.

By Messrs. William Harris and Co. 50, Holborn, London.

*From the 20th July to the 19th August, 1819, inclusive.*

| Day of Month. | Rain guage | Moon. | Therm. | | | Barom. | | De Luc's Hygrom. Dry | Damp | | Wind. | | Atmospheric Variation. | | |
|---|---|---|---|---|---|---|---|---|---|---|---|---|---|---|---|
| July | | | | | | | | | | | | | | | |
| 20 | ,15 | | 65 | 73 | 53 | 29·40 | 29·42 | | 11 | 11 | SSW | N | Fine | | Rain |
| 21 | ,20 | | 56 | 63 | 52 | 29·53 | 29·88 | | 12 | 8 | N va. | WNW | Cloud. | Sho'ry | |
| 22 | ,09 | ● | 58 | 65 | 55 | 30·00 | 30·08 | | 7 | 7 | N va. | N | Fine | | |
| 23 | | | 63 | 66 | 62 | 30·16 | 30·18 | | 8 | 8 | NNW | WSW | Fine | | |
| 24 | | | 68 | 76 | 60 | 30·20 | 30·22 | | 7 | 7 | N | SW | Fine | | |
| 25 | | | 70 | 79 | 58 | 30·12 | 30·03 | | 9 | 10 | SW | SSE | Fine | | |
| 26 | | | 67 | 73 | 58 | 30·04 | 30·10 | | 10 | 9 | E | E | Fine | | |
| 27 | | | 66 | 76 | 58 | 30·13 | 30·17 | | 10 | 9 | NE | ENE | Cloud. | Fine | |
| 28 | | | 65 | 76 | 58 | 30·16 | 30·17 | | 9 | 8 | NE | NE | Cloud. | Fine | |
| 29 | | | 64 | 76 | 59 | 30·16 | 30·12 | | 11 | 10 | NE | NE | Cloud. | Fine | |
| 30 | | ☾ | 70 | 82 | 63 | 30·10 | 30·06 | | 8 | 3 | NE | NE | Fine | | |
| 31 | | | 70 | 78 | 64 | 30·03 | 30·04 | | 9 | 9 | ENE | ENE | Fine | | |
| Aug. | | | | | | | | | | | | | | | |
| 1 | | | 71 | 76 | 60 | 30·05 | 30·02 | | 10 | 11 | NE | ESE | Cloud. | Rain | Cloud. |
| 2 | ,20 | | 63 | 71 | 61 | 30·00 | 29·92 | | 11 | 8 | ENE | NNE | Cloud. | | Fine |
| 3 | | | 63 | 70 | 61 | 29·92 | 29·93 | | 7 | 7 | NE | NE | Cloud. | Fine | |
| 4 | | | 65 | 69 | 6[illegible] | 29·91 | 29·92 | | 9 | 10 | N | N | Cloud. | | |
| 5 | | ○ | 64 | 67 | 61 | 29·93 | 30·00 | | 11 | 12 | N | N | Cloud. | | |
| 6 | | | 66 | 75 | 64 | 30·04 | 29·95 | | 9 | 12 | W | W | Cloud. | Fine | Cloud. |
| 7 | | | 70 | 78 | 62 | 33·03 | 30·03 | | 12 | 10 | NW | NE | Fine | | Cloud. |
| 8 | | | 67 | 70 | 61 | 30·09 | 30·12 | | 6 | 5 | N | N | Fine | | |
| 9 | | | 71 | 76 | 64 | 30·15 | 30·11 | | 5 | 7 | NE | ESE | Fine | Sho'ry | Fine |
| 10 | | | 70 | 75 | 58 | 30·09 | 30 05 | | 6 | 8 | ESE | SE | Fine | | |
| 11 | ,02 | | 61 | 76 | 61 | 30·00 | 29·95 | | 11 | 9 | ESE | SE | Fog | Cloud. | Fine |
| 12 | | ☽ | 64 | 75 | 65 | 29 95 | 29·92 | | 11 | 8 | SE | WSW | Cloud. | Fine | Cloud. |
| 13 | | | 70 | 78 | 6[illegible] | 29·95 | 30·00 | | 9 | 11 | W | WSW | Cloud. | Fine | Rain |
| 14 | | | 64 | 75 | 65 | 30·03 | 30·04 | | 8 | 7 | NE | N | Fine | | |
| 15 | ,03 | | 68 | 72 | 60 | 30·10 | 30·13 | | 7 | 5 | NE | N | Fine | | |
| 16 | | | 72 | 78 | 65 | 30·18 | 30·22 | | 8 | 7 | NE | NNE | Fine | | Cloud. |
| 17 | | | 68 | 77 | 6[illegible] | 30·23 | 30·30 | | 8 | 9 | NNE | NE | Fine | | Cloud. |
| 18 | | | 66 | 75 | 59 | 30·30 | 30·29 | | 11 | 12 | NE | NE | Cloud. | Fine | Cloud. |
| 19 | ,01 | | 65 | 70 | 60 | 30·22 | 30·20 | | 11 | 9 | ENE | ENE | Cloud. | | Fine |

The quantity of rain fallen in July, is 1 inch and 24-100ths.

